# Bio-Based Polyurethane Asphalt Binder with Continuous Polymer-Phase Structure: Critical Role of Isocyanate Index in Governing Thermomechanical Performance and Phase Morphology

**DOI:** 10.3390/molecules30112466

**Published:** 2025-06-04

**Authors:** Haocheng Yang, Suzhou Cao, Chengwei Wu, Zhonghua Xi, Jun Cai, Zuanru Yuan, Junsheng Zhang, Hongfeng Xie

**Affiliations:** 1 MOE Key Laboratory of High Performance Polymer Materials and Technology, School of Chemistry and Chemical Engineering, Nanjing University, Nanjing 210023, China; 522023240043@smail.nju.edu.cn (H.Y.); 522024240001@smail.nju.edu.cn (S.C.); 522022240039@smail.nju.edu.cn (C.W.); 2 Experimental Chemistry Teaching Center, School of Chemistry and Chemical Engineering, Nanjing University, Nanjing 210023, China; xizh@nju.edu.cn; 3 Public Instrument Center, School of Chemistry and Chemical Engineering, Nanjing University, Nanjing 210023, China; caijun@nju.edu.cn; 4 Modern Analysis Center, Nanjing University, Nanjing 210023, China; zryuan@nju.edu.cn

**Keywords:** polyurethane asphalt, bio-based polyurethane, isocyanate index, glass transition temperature, phase separation, mechanical properties

## Abstract

Polyurethane asphalt (PUA) has attracted considerable attention in the field of pavement engineering. However, traditional PUA systems typically exhibit low concentrations of polyurethane (PU), leading to a continuous bitumen-dominated phase that adversely affects mechanical properties. Furthermore, the non-renewable nature of raw materials raises environmental concerns. To address these limitations, this study developed an eco-friendly and cost-efficient bio-based PUA binder (PUAB) featuring a continuous high-biomass PU matrix (over 70% biomass) and a high bitumen content (60 wt%). The effects of the isocyanate index (NCO/OH ratio) on the cure kinetics, rheological behavior (rotational viscosity over time), viscoelasticity, damping capacity, phase morphology, thermal stability, and mechanical performance were systematically investigated using Fourier-transform infrared spectroscopy, dynamic mechanical analysis, laser-scanning confocal microscopy, and tensile testing. Key findings revealed that while the rotational viscosity of PUABs increased with a higher isocyanate index, all formulations maintained a longer allowable construction time. Specifically, the time to reach 1 Pa·s for all PUABs at 120 °C exceeded 60 min. During curing, higher isocyanate indices reduced final conversions but enhanced the storage modulus and glass transition temperatures, indicating improved rigidity and thermal resistance. Phase structure analysis demonstrated that increasing NCO/OH ratios reduced bitumen domain size while improving dispersion uniformity. Notably, the PUAB with the NCO/OH ratio of 1.3 achieved a tensile strength of 1.27 MPa and an elongation at break of 238%, representing a 49% improvement in toughness compared to the counterpart with an NCO/OH ratio = 1.1. These results demonstrate the viability of bio-based PUAB as a sustainable pavement material, offering a promising solution for environmentally friendly infrastructure development.

## 1. Introduction

Bitumen, also known as asphalt binder, is a critical viscoelastic adhesive in pavement engineering, significantly influencing the performance characteristics of bituminous mixtures despite its relatively low dosage (typically 4–7% by weight). However, its inherent temperature-dependent behavior presents challenges: it exhibits Newtonian fluidity at high temperatures and brittle fracture under low-temperature conditions. This leads to three primary failure modes: rutting deformation, thermal cracking, and fatigue damage. These performance limitations, which are further compounded by increasing traffic loads, climatic variations, and maintenance difficulties, underscore continuous improvement in bitumen modification technologies [1,2].

Polymer modification has emerged as the predominant strategy for asphalt enhancement [3,4]. Compared to thermoplastic modifiers, thermosetting polymers such as epoxy resin and polyurethanes (PU) demonstrate superior performance advantages due to their crosslinked network structures [5,6,7]. Notably, polyurethane asphalt (PUA) systems have attracted increasing attention because of their exceptional adhesion strength, thermal stability, and mechanical durability [8,9,10]. The tunable physicochemical properties of PU, governed by monomer stoichiometry (NCO/OH ratio) and segmental microphase separation characteristics [11,12], allow for precise performance customization. Recent studies have systematically investigated the effects of the isocyanate type [13], polyol molecular weight [14], and hard segment contents [15] on PUA performance metrics.

In the context of sustainable development, bio-based polyurethanes derived from renewable resources, such as castor oil (CO), have shown particular promise. CO-based PUs exhibit enhanced thermal stability (2 wt% loading) [16], improved aging resistance (3–9 wt% range) [17,18,19], and superior adhesion properties (+27.3% with 10 wt% lignin–PU) [20]. Nevertheless, existing bio-PUA formulations predominantly maintain bitumen as the continuous phase (≤7 wt% PU) [21], limiting their application in demanding scenarios, such as steel bridge deck pavements. Crucially, the stoichiometric influence of the NCO/OH ratios on bio-PUA performance remains unexplored.

This study addresses these research gaps by systematically investigating the effects of the isocyanate index (NCO/OH = 1:1.1–1.3) on bio-PU asphalt binders (PUABs) based on castor oil and isophorone diisocyanate (IPDI), with high-biomass PU-dominated matrices (over 70% biomass) and a high bitumen content (60 wt%). We employ multi-scale characterization techniques, including attenuated total reflectance Fourier-transform infrared (ATR-FTIR) spectroscopy, Brookfield rotational viscometer, differential scanning calorimetry (DSC), dynamic mechanical analysis (DMA), thermogravimetric analysis (TGA), universal test machine (UTM), and laser-scanning confocal microscopy (LCM), to elucidate the cure kinetics, rotational viscosity (RV)–time characteristics, thermomechanical behavior, and microstructures of bio-based PUABs.

## 2. Results and Discussion

### 2.1. Rotational Viscosity–Time Characteristics

Rotational viscosity represents a critical rheological parameter for thermosetting polymer-modified asphalts, distinguishing them from thermoplastic counterparts through their dual dependence on both temperature and curing time [22]. This time-temperature superposition effect arises from the progressive increase in molecular weight during polyaddition reactions between low-molecular-weight monomers or oligomers. The exothermic nature of these crosslinking reactions further accelerates viscosity development via thermal autocatalysis [8].

From an application standpoint, bitumen must maintain an RV of ≤3 Pa⸱s throughout the entire construction window, which includes mixing, transportation, paving, and compaction processes [23]. This operational constraint necessitates precise control over the cure kinetics of thermosetting systems.

**Figure 1:**
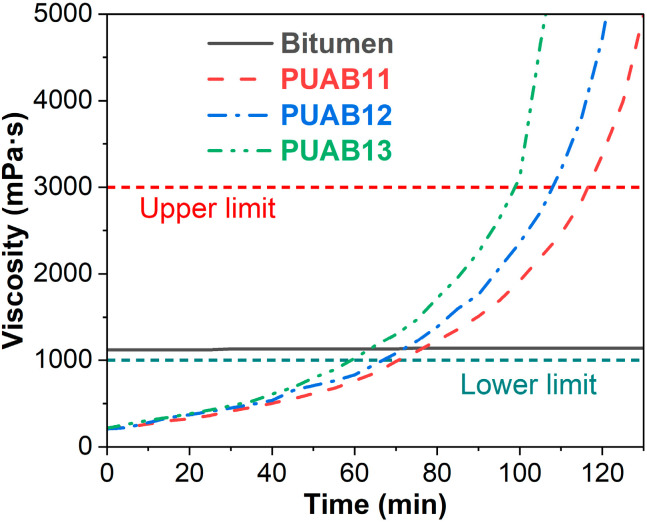
Rotational viscosity as a function of time for bitumen and PUABs at 120 °C.

Figure 1 demonstrates the isocyanate index-dependent evolution of RV for PUABs at 120 °C. Key observations include the following:

(1) A progressive increase in viscosity with curing time, reflecting continuous network formation.

(2) Enhanced reaction kinetics at higher isocyanate indices, as evidenced by steeper RV–time slopes.

Table 1 chronologically documents two critical rheological thresholds:

- Lower limit (time to reach 1 Pa·s): The minimum time for viscosity to reach 1 Pa·s, which is necessary for thermosetting polymer-modified asphalt materials;
- Upper limit (time to reach 3 Pa·s): The maximum allowable construction time for effective compaction.

Notably, PUAB systems exhibit extended operational windows compared to the conventional warm-mix epoxy asphalt binder (WEAB):

- 1 Pa⸱s attainment: 64–77 min (PUABs) versus 40 min specification (WEAB [24]);
- 3 Pa⸱s attainment: 99–117 min (PUABs) versus 70 min benchmark (WEAB [25]).

This delayed gelation behavior suggests superior processability of bio-based PUABs, potentially attributed to the following:

(1) Reduced reactivity of aliphatic isocyanates (e.g., IPDI) compared to aromatic counterparts [26];

(2) Steric hindrance effects in triglyceride-based polyols (CO) [27].

### 2.2. Structural Characterization

**Figure 2:**
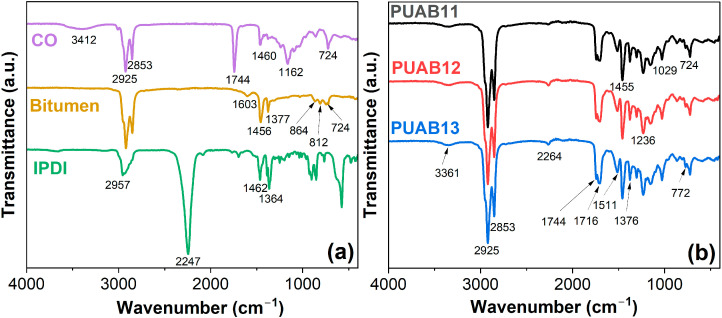
FTIR spectra of CO, bitumen, IPDI (**a**), and PUABs cured at 120 °C for 4 h (**b**).

Figure 2 presents the FTIR spectra of cured PUABs and their raw materials. The assignments of the characteristic peaks are summarized in Table 2. As depicted in Figure 2a, in the CO spectrum, the broad peak at approximately 3412 cm^−1^ is attributed to the –OH groups. The peaks at 2925 and 2853 cm^−1^ correspond to the asymmetric and symmetric C–H stretching vibrations of the –CH_2_ groups, respectively, while the peaks at 1744 and 1162 cm^−1^ are assigned to the C=O and C–O–C groups, respectively [28].

In the bitumen spectrum, the characteristic peaks at 1603, 864, and 812 cm^−1^ are associated with aromatic ring vibrations. Additionally, peaks at 1456 cm^−1^ (scissoring vibration of –CH_2_–), 1377 cm^−1^ (umbrella vibration of –CH_3_), and 724 cm^−1^ (sympathetic vibration of [–CH_2_–]n, where *n* ≥ 4) indicate the presence of aliphatic structures [29].

In the IPDI spectrum, the sharp peak at 2247 cm^−1^ corresponds to the –NCO group, while peaks at 1462 and 1364 cm^−1^ arise from –CH– deformation and –CH_2_ bending vibration, respectively [30].

After curing at 120 °C for 4 h (Figure 2b), the intensity of the –NCO group at 2264 cm^−1^ decreases significantly due to the reaction of isocyanate groups (IPDI) and hydroxyl groups (CO), leading to the formation of urethane bonds (–NH–CO–O). The key absorption bonds at 3361 cm^−1^ (–NH stretching), 1716 cm^−1^ (amide I: C=O stretching), 1511 cm^−1^ (–N–H in-plane bending), and 772 cm^−1^ (N–H out-of-plane bending) confirm the formation of polyurethane networks [31,32].

### 2.3. Cure Kinetics

**Figure 3:**
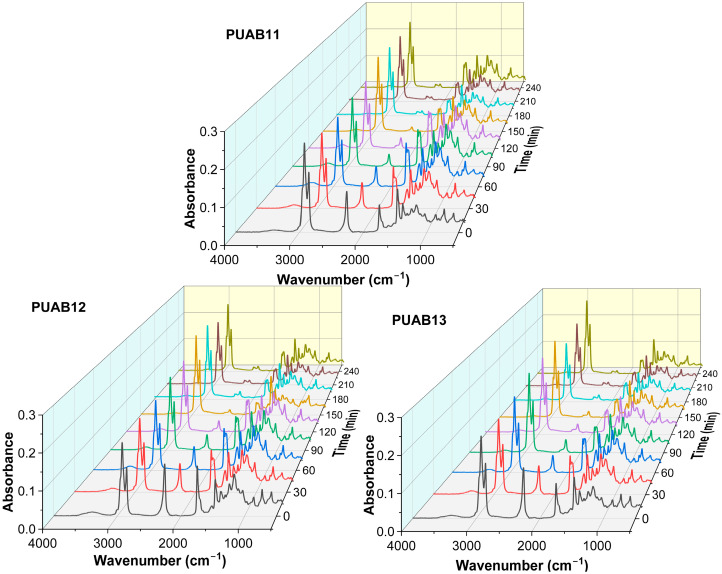
FTIR spectra of PUABs during curing at 120 °C.

FTIR serves as an effective tool for monitoring the curing process of polyurethane, as the absorbance of the –NCO and –OH groups during curing follows the Beer–Lambert law [33]. The conversion was determined by tracking the reduction in isocyanate groups in FTIR spectra (Figure 3) during curing at 120 °C, as calculated using Equation (1) [34]:

(1) $$\alpha =\left(1-\frac{A_{I,t}/A_{R,t}}{A_{I,0}/A_{R,0}}\right)\times 100\%$$

where A_I,t_ and A_I,0_ represent the absorbance intensities of the –NCO groups at 2264 cm^−1^ at the curing time (t) and initial time (0), respectively. A_R,t_ and A_R,0_ denote the absorbance intensities of the internal reference peak at 2925 cm^−1^ at the corresponding times.

**Figure 4:**
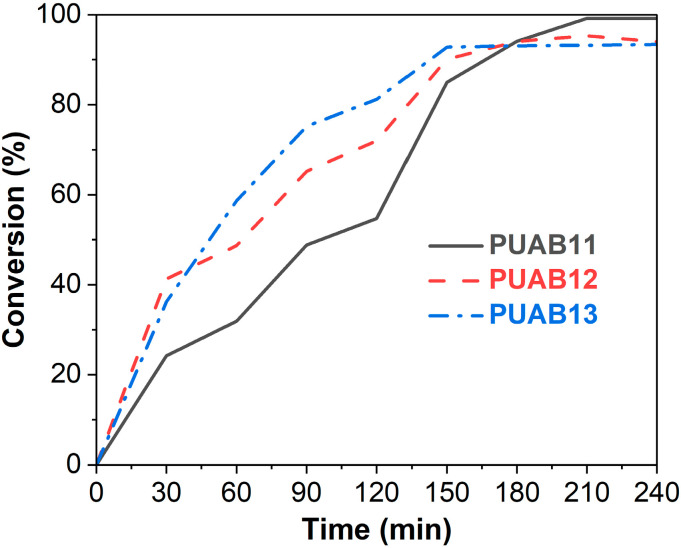
Conversions of PUAB during curing at 120 °C.

Figure 4 presents the conversions of PUABs as a function of curing time at 120 °C. The conversion of PUABs exhibits a positive correlation with the NCO/OH molar ratio during the first 180 min of curing; however, this trend reverses after 180 min. Upon completion of the 240 min curing process, PUAB11, PUAB12, and PUAB13 achieve conversions of 99%, 94%, and 93%, respectively. Liu et al. [35] explored the impact of the isocyanate index on the conversion in bio-based PU sealants, demonstrating that higher isocyanate indices correlate with reduced conversions throughout the curing process.

Thermosetting polymers form crosslinked three-dimensional (3D) networks through the curing of low-molecular-weight monomers or multifunctional prepolymers (oligomers) (functionality > 2). The curing mechanism progresses through two distinct stages: chemically controlled and diffusion-controlled [36]. During the chemically controlled stage, the polymerization of low-molecular-weight monomers or oligomers induces progressive branching and molecular-weight growth until gelation occurs, forming an immobile and insoluble network. Subsequent crosslinking density enhancement continues until the glass transition temperature of the material approaches the curing temperature, triggering vitrification. After vitrification, the reaction kinetics transition to diffusion control, significantly slowing down and ultimately terminating prior to full conversion [37]. To overcome vitrification and complete curing, a post-curing step at elevated temperatures is typically required.

In a chemically controlled regime (0–180 min), the PUAB conversion depends on the intrinsic reaction rate of polyurethane. This explains the observed positive correlation between the isocyanate index and conversion, consistent with the rotational viscosity versus time profile shown in Figure 1. Conversely, during the diffusion-controlled stage (>180 min), a higher initial conversion of PUAB systems results in increased steric hindrance, leading to reduced ultimate conversions after 4 h of curing, as illustrated in Figure 4.

### 2.4. Glass Transition Temperature (T_g_)

The glass transition refers to the transformation of bitumen from a glassy state to a viscoelastic state, accompanied by significant changes in its mechanical and thermodynamic properties [38]. This transition temperature (*T*_g_) serves as a critical parameter for characterizing bitumen’s elasticity, stiffness, and flow behavior [39,40]. Excessively low *T*_g_ values may cause softening/deforming at elevated temperatures, while an overly high *T*_g_ could lead to low-temperature brittleness and an increased susceptibility to cracking.

**Figure 5:**
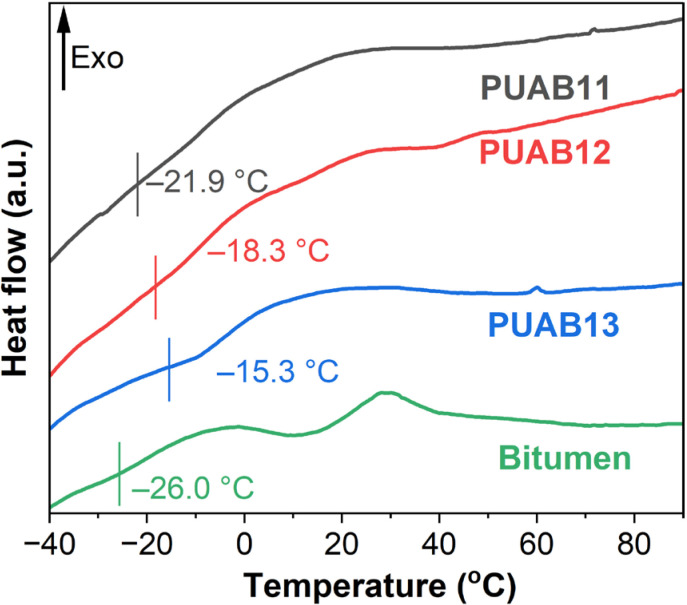
DSC curves of bitumen and PUABs.

In this study, both DSC and DMA were employed to determine the *T*_g_ of PUABs. Figure 5 presents the DSC curves, revealing a single *T*_g_ near −20 °C for all PUAB formulations, approximately 6 °C higher than that of pristine bitumen (−26.0 °C). This indicates that bio-based polyurethane elevates the *T*_g_ of bitumen. Quantitatively, the *T*_g_ values show a positive correlation with the isocyanate index (Table 3).

**Figure 6:**
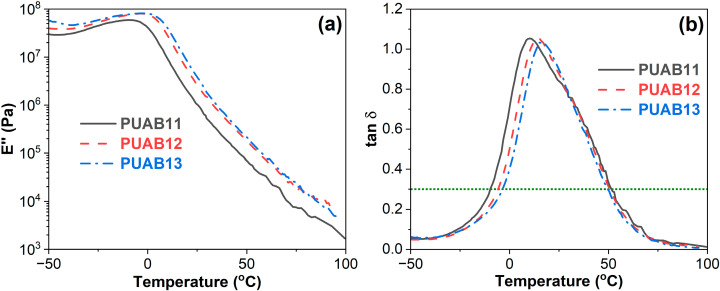
Loss modulus—(**a**) and damping factor—temperature curves (**b**) of PUABs.

DMA measurements provide complementary insights through the temperature-dependent profiles of the storage modulus (E′), loss modulus (E″), and loss factor or damping factor (tan δ) [41]. Consistent with established practice, the peak temperatures of both E″ and tan δ curves were adopted as *T*_g_ indicators. As illustrated in Figure 6, these characteristic temperatures systematically shift to higher values with an increasing isocyanate index, aligning well with the *T*_g_ trends derived from DSC (Table 3). Notably, all PUABs exhibit a single *T*_g_, attributed to the close proximity of the *T*_g_ values between bitumen and the polyurethane derived from castor oil and isophorone diisocyanate [31,42]. This contrasts with epoxy asphalts, which typically display two distinct *T*_g_ values corresponding to their immiscible bitumen and cured epoxy phases [43]. The inherent phase separation in epoxy asphalt systems, arising from the significant *T*_g_ disparity between epoxy resin (>25 °C) and bitumen, predisposes these materials to fatigue cracking during long-term service—a limitation effectively mitigated in the PUAB system.

### 2.5. Storage Modulus

**Figure 7:**
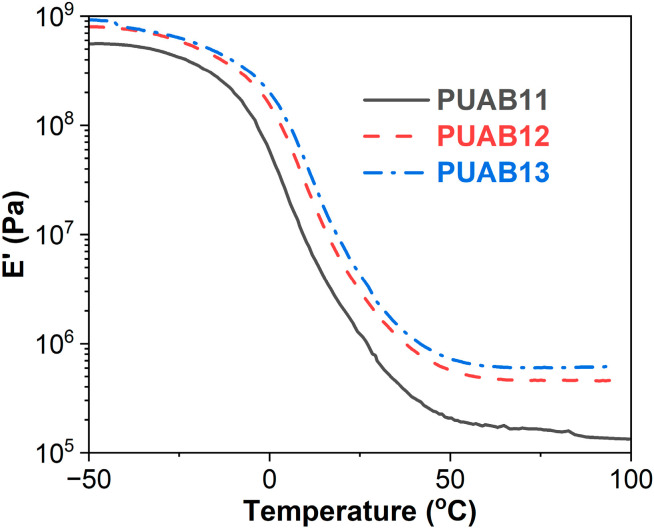
Storage modulus–temperature curves of PUABs.

Figure 7 illustrates the storage modulus of PUABs as a function of temperature. Throughout the measured temperature range (−50 to 100 °C), a consistent trend emerges where the E′ of PUABs increases with an elevating isocyanate index, demonstrating enhanced material stiffness at higher isocyanate index levels.

The *E′* value at rubbery plateau (*E′*_R_) serves as a critical parameter for evaluating the crosslinking density (**υ**_e_) through rubber elasticity theory [44]:

(2) $$\nu_{e}=\frac{E_{R}^{\prime }}{3RT_{R}}$$

where *T*_R_ denotes the absolute temperature in the rubbery state (*T*_g_ + 40 K). R represents the gas constant. As quantified in Table 3, the crosslinking density of PUABs exhibits a progressive increase with the isocyanate index. This trend directly correlates with the previously observed enhancement in the *T*_g_ values. Furthermore, the presence of functional groups in bitumen, including hydroxyl, thiol, and carboxylic acid moieties [45], enables chemical reactions with isocyanate groups [46,47]. These interfacial interactions contribute to the formation of additional crosslinks, thereby amplifying both the crosslinking density and *T*_g_ values in PUAB systems.

### 2.6. Damping Properties

Polyurethane and bitumen exhibit outstanding damping abilities, making them widely utilized in vibration and noise reduction devices [48,49]. In DMA, the damping characteristics of polymeric materials are typically characterized by a temperature-dependent loss modulus and damping factor curves [50,51].

As summarized in Table 4, all PUAB specimens demonstrate (tan δ)_max_ (the maximum value of tan δ) exceeding 1.00, confirming their superior energy dissipation capacity through viscoelastic mechanisms involving internal friction and molecular chain rearrangements. Moreover, these modified binders exhibit a broad effective damping range (**Δ**T, defined as the temperature interval with tan δ > 0.3) and substantial integrated damping (A_tanδ-T_). These collective metrics establish PUABs as high-performance damping materials.

Notably, the damping parameters show a progressive reduction with an increasing isocyanate index. This inverse correlation suggests that enhanced crosslinking density (as discussed in Section 2.5) compromises the molecular mobility required for optimal energy dissipation, thereby diminishing the damping efficiency of the composite system.

### 2.7. Cole–Cole Plots

**Figure 8:**
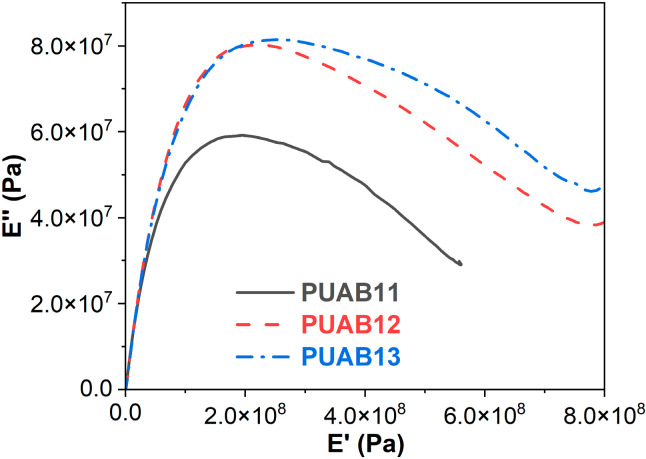
Cole–Cole plots of PUABs.

Figure 8 depicts the E″ versus E′ relationships (Cole–Cole plots) of PUABs. A singular semicircular arc is observed across all PUABs’ Cole–Cole profiles, signifying homogeneous phase compatibility between the castor oil-based polyurethane and bitumen components. This monomodal relaxation behavior contrasts sharply with epoxy asphalt systems reported in the literature [52], where bimodal arcs (corresponding to distinct epoxy and bitumen phases) are evident due to the thermodynamic immiscibility of the constituent materials.

### 2.8. Morphology

Due to the insoluble and infusible characteristics of thermosetting polymers, they are immiscible with bitumen, although full or partial compatibility may exist between uncured thermoset precursors and bitumen during the initial curing stage [53]. This inherent immiscibility leads to inevitable phase separation in all thermosetting polymer-modified asphalt systems. The formation of continuous/discontinuous phases is governed by the ratio of thermosetting polymer to bitumen [54,55].

**Figure 9:**
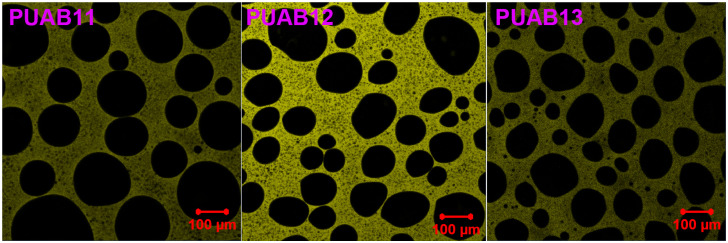
Phase morphology images of cured PUABs.

Figure 9 illustrates the phase morphology of cured PUABs observed through LCM. Notably, black bitumen domains are uniformly dispersed within the continuous PU matrix, forming the discontinuous phase in all PUAB samples. This phenomenon persists even at a 60 wt% bitumen content—a threshold where phase inversion (transition to bitumen-continuous structure) typically occurs in epoxy asphalt systems [56]. The observed phase continuity of PU in high-bitumen-content composites may stem from enhanced compatibility between plant oil-derived polyols and bitumen components [22,57].

**Figure 10:**
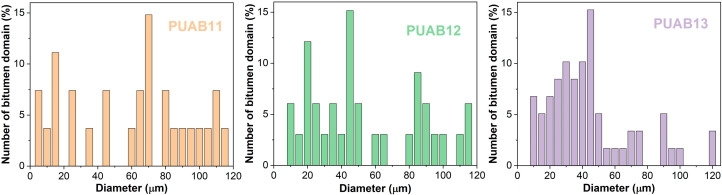
Particle size distribution of dispersed bitumen domains in cured PUABs.

To quantitatively analyze the microstructural evolution, Image-Pro Plus 6.0 software was employed to determine the average particle size and size distribution of bitumen domains. The number-average (*D*_n_) and weight-average (*D*_w_) diameters were calculated using Equations (3) and (4):

(3) $$D_{n}=\frac{\Sigma n_{i}D_{i}}{\Sigma n_{i}}$$

(4) $$D_{w}=\frac{\Sigma n_{i}{D_{i}}^{2}}{\Sigma n_{i}D_{i}}$$

where *n*_i_ represents the number count of domains with diameter *D*_i_. Figure 10 illustrates the particle size distribution of dispersed bitumen domains in cured PUABs. Table 5 summarizes the *D*_n_, *D*_w_, and dispersity index (Ɖ = *D*_w_/*D*_n_) values. Both average diameters and Ɖ values decrease systematically with an increasing isocyanate index, indicating that (1) enhanced crosslinking density restricts domain growth and (2) improved dispersion uniformity within the polymer index.

### 2.9. Thermal Stability

**Figure 11:**
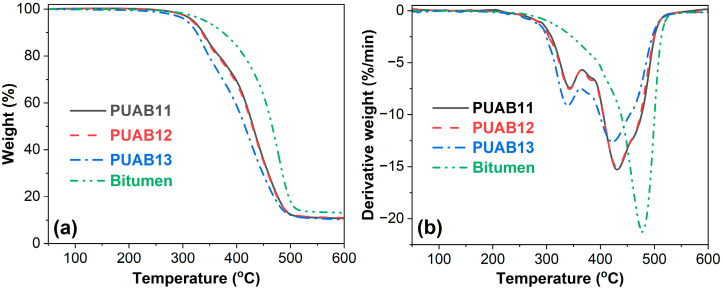
TGA (**a**) and DTG (**b**) curves of PUABs.

Figure 11 depicts the weight loss and derivative weight loss curves of bitumen and PUABs as functions of temperature, with quantitative data summarized in Table 6. The initial degradation temperature at a 5% weight loss (*T*_i_) of PUABs is significantly lower than that of neat bitumen and decreases with an increasing isocyanate index, indicating that a higher NCO/OH ratio adversely affects the thermal stability of PUABs. This observation aligns with previous reports on PU sealants [35]. Notably, the *T*_i_s of PUABs remain substantially higher than those of castor oil-based PUs, owing to the stabilizing effect of bitumen [31].

Interestingly, contrary to PUABs, pure PUs exhibit improved thermal stability with an increasing isocyanate index. As shown in the derivative thermogravimetry (DTG) profiles (Figure 11b), neat bitumen displays a single dominant degradation step. In contrast to the three-stage degradation behavior reported for pure CO/IPDI-based PUs [31,58], PUABs exhibit only two distinct degradation stages, likely due to a chemical interaction between isocyanate groups in the PU matrix and functional groups within bitumen. The first degradation stage accounts for approximately 25% of the total weight loss, while the second stage corresponds to the remaining 75%.

As illustrated in Table 6, both of the maximum degradation rate temperatures at the first and second steps (*T*^1^_dmax_ and *T*^2^_dmax_) of PUABs decrease with an increasing NCO/OH ratio, further confirming the progressive deterioration of thermal stability at higher isocyanate indices. From the perspective of bitumen modification, these results imply that incorporating bio-based PU reduces the inherent thermal stability of bitumen. Although the bitumen content in PUABs remains constant at 60 wt%, the extent of interfacial interaction between the bitumen functional groups and the isocyanate groups is governed by the concentration of isocyanate groups. Specifically, higher NCO/OH ratios increase the density of reactive isocyanate groups, thereby intensifying bitumen–polyurethane interactions and paradoxically accelerating thermal degradation.

Additionally, Table 6 reveals that the char residue at 600 °C for PUABs is lower than that of neat bitumen, attributed to the interfacial interaction between bitumen and PU. The incorporation of bio-based PU lowers the char residue. However, no significant correlation is observed between the isocyanate index and the char residue content.

### 2.10. Mechanical Performance

**Figure 12:**
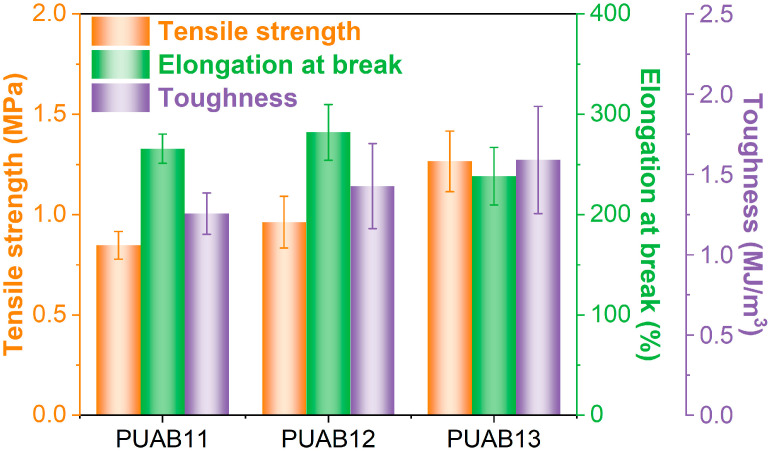
Mechanical properties of PUABs.

The mechanical properties of PUABs, as determined from stress–strain uniaxial tests, are presented in Figure 12. The tensile strength of PUABs increases with the isocyanate index, which can be attributed to the enhanced crosslinking density at higher NCO/OH ratios (Table 3). Specifically, the tensile strengths of PUAB12 and PUAB13 are 14% and 49%, respectively, higher than that of PUAB11. The elongation at break of PUABs initially increases with the isocyanate index, reaching a maximum value for PUAB12, but subsequently decreases at higher NCO/OH ratios. Similar trends in tensile strength and elongation at break have been reported in polyurethane sealant systems [35]. Notably, the elongation at break of all PUAB samples exceeds the requirement for warm-mix epoxy asphalt binders (>200%). However, the maximal tensile strength of PUAB13 (1.27 MPa) remains slightly below the WEAB requirement (1.50 MPa) [24].

The area under the stress (σ) versus strain (ε) curve in uniaxial tension represents the material toughness (τ), corresponding to the total energy required for fracture [59]:

(5) $$\tau =\int_{0}^{\varepsilon_{b}}\sigma d\varepsilon$$

where ε_b_ denotes the elongation at break. Consistent with the trend in tensile strength, the toughness of PUABs is enhanced with an increasing isocyanate index. The toughness values of PUAB12 and PUAB are 14% and 27%, respectively, higher than those of PUAB11, reflecting the continuous increase in tensile strength.

## 3. Materials and Methods

### 3.1. Materials

Bitumen of a 60/80 penetration grade was sourced from China Offshore Bitumen (Taizhou) Co., Ltd. (Taizhou, China), with its key physicochemical characteristics detailed in Table 7. CO was procured from Bide Pharmtech Co., Ltd. (Shanghai, China). IPDI (industrial grade) was obtained from Yantai Wanhua Polyurethanes Co., Ltd. (Yantai, China).

### 3.2. Preparation of Bio-Based PUABs

**Figure 13:**
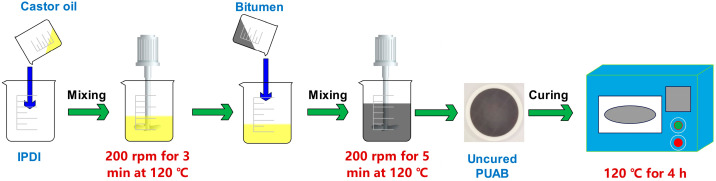
Schematic presentation of PUAB preparation.

As illustrated in Figure 13, the synthesis process involved the following steps:

(1) CO and IPDI were initially mixed at 120 °C for 3 min at a rotation speed of 200 rpm.

(2) Preheated bitumen (120 °C, conditioned for 30 min) was then incorporated into the CO-IPDI mixture.

(3) After 5 min of continuous mixing at 120 °C (200 rpm), the uncured PUAB composite was obtained.

(4) The composite was transferred into circular Teflon molds (100 mm diameter × 5 mm height) and subsequently cured in an oven at 120 °C for 4 h.

All formulations maintained a fixed bio-based polyurethane-to-bitumen weight ratio of 40:60. The prepared specimens were designated as PUAB11, PUAB12, and PUAB13, corresponding to NCO/OH molar ratios of 1:1.1, 1:1.2, and 1:1.3 in the polyurethane formulation, respectively. Table 8 illustrates the formulations of PUABs and the biomass content of bio-PU used. The biomass contents of all bio-PUs exceeded 70%.

### 3.3. Methods

#### 3.3.1. ATR-FTIR Spectroscopy

ATR-FTIR spectroscopy was performed using a Bruker Alpha spectrometer (Bruker, Ettlingen, Germany) equipped with a single-bounce diamond ATR accessory. Spectral data were acquired in the 4000–400 cm^−1^ range with a resolution of 4 cm^−1^ and 16 scan accumulations. The continuous monitoring of curing was implemented during the 120 °C curing process, with spectra recorded at 30 min intervals throughout the 240 min experimental duration.

#### 3.3.2. Rheological Behavior

Rotational viscosity measurements were conducted on a Changji NDJ-1 viscometer (Shanghai, China), following the ASTM D4402-06 standard [63]. Using spindle #18 at a 50 rpm rotational speed, the viscosity was monitored at 120 °C until it reached 5 Pa⸱s.

#### 3.3.3. DSC

**Figure 14:**
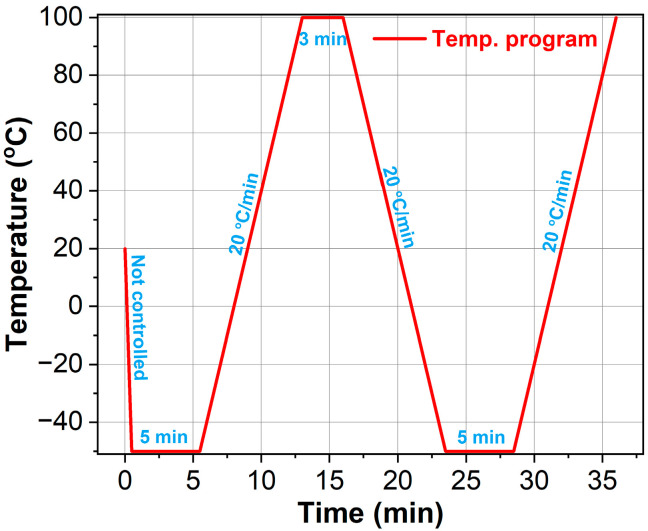
DSC temperature cycle for determining the *T*_g_s of PUABs.

Differential scanning calorimetry was performed using a Pyris 1 instrument (Perkin-Elmer, Norwalk, CT, USA) under a continuous argon flow of 20 mL/min. Polyurethane-based samples (PUABs, ~5 mg) were sealed in aluminum crucibles and subjected to heating/cooling cycles at a constant rate of 20 °C/min. The thermal protocol (Figure 14) comprised three sequential steps:

(1) Initial heating and cooling: Samples were rapidly cooled to −50 °C (maximum instrument rate) followed by a 5 min isothermal hold to erase thermal history (chemical/physical aging effects).
(2) Equilibration: Temperature stabilization was achieved prior to the second heating phase.
(3) Second heating: Glass transition temperatures were determined via heat flow analysis.

#### 3.3.4. DMA

Dynamic mechanical analysis was conducted in tension mode using a DMA + 450 instrument (01 dB-Metravib, Limonest, France) to evaluate the viscoelastic properties of PUABs. Temperature-sweep measurements were performed over the range of −50 °C to 100 °C at 3 °C intervals, with a fixed oscillatory frequency of 1 Hz.

#### 3.3.5. TGA

Thermogravimetric analysis was performed on a TGA/DSC1 instrument (Mettler Toledo, Zurich, Switzerland) to assess the thermal stability of PUABs under a nitrogen purge (20 mL/min). Approximately 10 mg of sample was loaded into an alumina crucible and heated from 50 °C to 600 °C at a constant heating rate of 20 °C/min.

#### 3.3.6. UTM

Uniaxial tensile testing was conducted using a universal testing machine (Instron 3366, Instron, Norwood, MA, USA) in accordance with the ASTM D638 standard [66] (Type V specimen geometry). Five dog bone-shaped specimens per PUAB formulation were tested at ambient temperature (23 ± 2 °C) with a constant crosshead displacement rate of 200 mm/min. The mean values from five replicates were calculated to ensure experimental repeatability.

#### 3.3.7. LCM

Laser scanning microscopy analysis was performed using a Zeiss LSM710 system (Zeiss, Jena, Germany) equipped with a 488 nm argon-ion excitation source. For microscopic observation, an uncured PUAB precursor was deposited onto preheated glass slides mounted on a temperature-controlled hot stage (120 °C). The liquid film was manually spread to achieve uniform thickness, followed by immediate covering with a cover slip. Specimens were subsequently transferred to a convection oven and thermally cured at 120 °C for 4 h under atmospheric conditions.

## 4. Conclusions

This study systematically investigated the effects of the isocyanate index on the performance and morphology of bio-based polyurethane asphalt binder featuring a continuous polyurethane phase. Key findings are summarized as follows:

(1) The rotational viscosity of PUABs increases proportionally with the isocyanate index. Although PUABs demonstrate longer allowable construction time compared to WEABs, elevated NCO/OH ratios progressively reduce these processing windows.
(2) PUABs exhibit a single glass transition temperature, confirming superior compatibility between bitumen and bio-based polyurethane. Notably, the *T*_g_ values of PUABs show a positive correlation with the isocyanate index, consistently exceeding that of neat bitumen.
(3) While increasing the isocyanate index negatively impacts the final conversion of the cure reaction, thermal stability, and damping properties, it significantly enhances the storage modulus and mechanical performance.
(4) Phase separation analysis reveals that higher NCO/OH ratios reduce the average diameters of dispersed bitumen domains (*D*_n_ from 59.9 μm to 45.0 μm and *D*_w_ from 85.6 μm to 58.3 μm) but concurrently increase domain size uniformity.
(5) The rotational viscosity–time profile and elongation at break (>230%) meet the requirements for the thermosetting binder in steel deck bridge pavements. However, suboptimal tensile strength (1.27 MPa versus the required 1.50 MPa) necessitates further formulation.
(6) Compared to conventional petroleum-based polyurethane asphalts, the biomass content of PU used for the production of bio-based PUAB containing 60 wt% bitumen is more than 70%, indicating that these PUABs are environmentally friendly and cost-efficient.

## Figures and Tables

**Table 1:** Time for the rotational viscosity of PUABs to reach 1 and 3 Pa⸱s.

Sample	Time to Reach 1 Pa⸱s (min)	Time to Reach 3 Pa⸱s (min)
PUAB11	77	117
PUAB12	72	108
PUAB13	64	99

**Table 2:** ATR-FTIR characteristic peak assignments for PUAB and its raw materials.

Peak Position (cm^−1^)	Characteristic Absorption Bands
3412	–OH group
3361	Stretching vibration of –NH
2957, 2925	Stretching –CH vibration of –CH_2_
2853	Symmetric stretching of –CH_2_
2247, 2264	–NCO group
1744	C=O group for ester
1716	Amide I: C=O stretching vibrations
1603, 854, 812	Vibration of aromatic rings
1511	–N–H in-plane bending
1462	Deforming vibrations of –CH–
1456	Scissoring vibration of –CH_2_–
1377	Umbrella vibration of –CH_3_
1364	Bending vibration of –CH_2_
1162	C–O–C group
772	N–H out-of-plane bending
724	Sympathetic vibration of [–CH_2_–]n, *n* ≥ 4

**Table 3:** *T*_g_s and crosslinking densities of PUABs.

Sample	*T*_g_ (°C)			υ_e_ (mol/m^3^)
	DSC	E″-T	tan δ-T	
PUAB11	−21.9	−8.5	10.2	25.7
PUAB12	−18.3	−2.6	14.6	63.3
PUAB13	−15.3	−1.7	16.1	78.9

**Table 4:** Damping parameters of PUABs.

Sample	(tan δ)_max_	ΔT (°C)	A_tanδ-T_ (K)
PUAB11	1.05	61.5 (−9.9~51.6)	52.7
PUAB12	1.05	56.6 (−5.9~50.7)	48.5
PUAB13	1.03	53.4 (−3.6~49.8)	45.7

**Table 5:** Average diameters and dispersity of bitumen domains in cured PUABs.

Sample	*D*_n_ (μm)	*D*_w_ (μm)	Ɖ
PUAB11	59.9 ± 2.6	85.6 ± 5.6	1.43
PUAB12	56.4 ± 4.7	74.5 ± 6.5	1.32
PUAB13	45.0 ± 6.7	58.3 ± 7.2	1.29

**Table 6:** TGA and DTG results of PUABs.

Sample	*T*_i_ (°C)	*T*^1^_dmax_ (°C)	*T*^2^_dmax_ (°C)	Char Residue at 600 °C (%)
PUAB11	319.6	344.1	431.6	10.7
PUAB12	316.9	343.0	431.0	11.0
PUAB13	306.9	339.1	423.1	10.3
Bitumen	341.8	477.3	-	13.1

**Table 7:** Physicochemical characteristics of bitumen.

Properties	Standard	Value
Physical properties		
Penetration (25 °C, 0.1 mm)	ASTM D5-06 [60]	73.0
Ductility (10 °C, cm)	ASTM D113-07 [61]	15.8
Softening point (°C)	ASTM D36-06 [62]	48.2
Viscosity (60 °C, Pa⸱s)	ASTM D4402-06 [63]	173.0
Wax content (%)	ASTM D3344-90 [64]	1.83
Chemical components		
Saturates (%)	ASTM D4124-09 [65]	20.0
Aromatics (%)		31.5
Resins (%)		37.1
Asphaltenes (%)		6.8

**Table 8:** Formulations of PUABs and biomass contents in bio-PUs.

**Sample**	**CO (g)**	**IPDI (g)**	**Bitumen (g)**	**Biomass Contents in Bio-PUs (%)**
PUAB11	100	35.0	202.5	74.1
PUAB12	100	38.7	208.0	72.1
PUAB13	100	41.9	212.9	70.5

## Data Availability

All data are available in the manuscript.
